# Mesoporous bimetallic Fe/Co as highly active heterogeneous Fenton catalyst for the degradation of tetracycline hydrochlorides

**DOI:** 10.1038/s41598-019-52013-y

**Published:** 2019-11-01

**Authors:** Junchao Li, Xuhua Li, Jindong Han, Fansheng Meng, Jinyuan Jiang, Jiao Li, Chunlian Xu, Yun Li

**Affiliations:** State Key Laboratory of Environmental Criteria and Risk Assessment, Chinese Research Academy of Environmental Sciences, Beijing, 100012 China

**Keywords:** Environmental sciences, Environmental sciences, Environmental sciences, Environmental chemistry, Environmental chemistry

## Abstract

Mesoporous bimetallic Fe/Co was prepared as a Fenton-like catalyst to degrade the tetracycline hydrochlorides (TC). The nanocasting strategy with KIT-6 as a hard template was carried out to synthesize the mesoporous bimetallic catalyst. The mesoporous bimetallic Fe/Co catalyst was characterized by X-ray diffraction (XRD), transmission electron microscopy (TEM), nitrogen adsorption-desorption isotherms, and Brunauer-Emmett-Teller (BET) method. The results showed that the catalyst has significant nanofeatures; the surface area, pore size, and particle size were 113.8 m^2^g^−1^, 4 nm, and 10 nm, respectively. In addition, the effects of the operating parameters, such as the iron-to-cobalt ratio, pH, H_2_O_2_, and initial TC concentrations on its catalytic performance were investigated. The best operating parameters were as follows: iron-to-cobalt ratio = 2:1 to 1:1, pH = 5–9, H_2_O_2_: 30 mmol, initial TC less than 30 mg/L. Furthermore, the mesoporous bimetallic Fe/Co showed a good performance for degrading TC, achieving a removal rate of 86% of TC after 3 h under the reaction conditions of H_2_O_2_ = 30 mmol, mesoporous bimetallic Fe/Co = 0.6 g/L, TC = 30 mg/L, pH = 7.0, and temperature = 25.5 °C. The mesoporous bimetallic Fe/Co catalyst shows good stability and reusability. This work demonstrated that mesoporous bimetallic Fe/Co has excellent catalytic efficiency, smaller amounts of leached ions, and wider pH range, which enhance its potential applications.

## Introduction

Antibiotics constitute a new class of water contaminants of emerging concern with adverse effects on the ecological environment^1^ as larger amounts of antibiotics are frequently detected in surface water^2^ and municipal sewage treatment plants^3^. Antibiotics are difficult to be treated by microorganisms and physical adsorption; in fact, only 20–30% of antibiotics can be biodegraded by microorganisms^4^ and removed by physical adsorption, resulting in complicated post-treatment problems of sorbents^5^. Advanced oxidation technologies (AOTs), based on the generation of highly reactive hydroxyl (•OH) free radicals, are used in wastewater treatment plants to remove various pollutants. They are simple processes with low energy consumption and without any secondary pollution and thus considered to be promising strategies^6–8^.

As one of the AOTs, heterogeneous catalysts have displayed satisfactory performance in Fenton-like processes; the pH usage limits are further alleviated without the precipitation of iron hydroxide^9^. Iron minerals possess the advantages of the low band gap (2.2 eV), low cost, and nontoxicity^10^. However, decreased degradation rates and large consumption of H_2_O_2_ limit the practical applications of heterogeneous Fenton-like catalysts^11,12^. The heterogeneous Fenton catalyst efficiency can be improved by increasing the surface area, enhancing their dispersion, and introducing suitable transition metals into heterogeneous catalysts^13–16^. Nanocatalysis is one of the many practical applications of nanotechnology^17–19^. Development of a nanocatalyst with a high surface area can accelerate Fenton-like reactions because the active sites are located on the surface and the catalytic performance of the nanocatalyst depends on the effect of “smaller particle size, higher activity^20,21^”.

Due to the flexible synthesis method, unique physical properties, easy surface functionalization, and good biocompatibility, mesoporous nanomaterials are increasingly used in adsorption, separation, catalysis, energy storage, and biomedical applications, especially as multi-functional drug delivery carriers for loading various chemical drugs, biomacromolecules, genes, and as a multifunctional diagnostic platform for composite magnetic properties^22–25^. In addition, the heterogeneous catalysts with transition metals improve not only the catalytic performance but also the reaction conditions under the synergistic effect of multimetals^16,20,21,26^.

This study aims to use KIT-6 as a template to prepare highly ordered mesoporous iron-cobalt heterogeneous catalysts^12^. The strategy of nanocasting (by solid phase method) accelerates the Fenton-like reaction and improves reaction conditions. Additionally, a new mechanism was proposed based on the experimental data analysis and literature review. Finally, the stability and reusability of the catalyst were evaluated.

## Methods

### Reagents

Ferric nitrate (Fe(NO_3_)_3_·9H_2_O), cobalt nitrate (Co(NO_3_)_2_·6H_2_O), butyl alcohol, hydrochloric acid (HCl), sodium hydroxide (NaOH), tetraethylorthosilicate (TEOS), and ethanol for preparation of ordered meso-Fe/Co were purchased from Sinopharm Chemical Reagent Co., Ltd., SCRC, China. H_2_O_2_ (30% v/v), ethanol, isopropanol, and tetracycline (TC) hydrochloride were purchased from Aladdin and triblock copolymer Pluronic P123 (Mw = 5800, EO20PO70EO20) from Sigma–Aldrich. The purity of the agents used was no lower than analytical grade, and no further purification work was performed on the agents. Deionized water was used for all experiments.

### Preparation of meso-Fe/Co

Meso-Fe/Co catalyst was prepared by template synthesis, and template material KIT-6 was synthesized by classical method^27,28^. Co(NO_3_)_2_·6H_2_O, Fe(NO_3_)_3_·9H_2_O and KIT-6 powder are evenly mixed, and ground using an and appropriate amount of ethanol is added to dissolve iron salt and cobalt salt. The mixture was soaked in ethanol solution for 12 hours to fully immerse the ethanol salt solution into KIT-6. The mixed solution was transferred to the water bath pot for evaporation, the obtained solid sample was further transferred to the tube furnace for calcining at 600 °C for 5 hours. The sample was cooled to room temperature and transferred to 2M NaOH solution for 24 hours under the condition of stirring, and then was further filtered, washed and dried. Finally, the reduction was conducted in a 450 °C muff furnace for 2 hours with the heating rate of 5° Cmin^−1^ in hydrogen atmosphere.

### Experimental procedure

Catalytic performance of mesoporous Fe/Co was tested at room temperature by using a constant temperature shaker (200 r/min) and a flask (1000 mL). The reaction system consists of catalyst (0.6 g/L), target contaminant (TC = 30.0 mg/L, volume = 200 mL, pH7.0 ± 0.5) and oxidant (H_2_O_2_, 3.0 mL). The reaction time was set at 180 minutes, and the pH value was adjusted by using HCl (0.5 M) and NaOH (0.5 M). Three parallel experiments were carried out for this study. The prepared catalysts were collected and stored in nitrogen environment, and their structures were characterized by SEM, TEM, XRD and BET. The reusability of the catalyst was evaluated by collecting the catalyst with magnet and reusing the catalyst for the next reaction under similar experimental conditions.

### Sample analysis

Concentration analysis test of tetracycline hydrochloride (TC) during catalytic reaction: Samples with different reaction times were filtered by 0.45 μm nylon membrane and tetracycline hydrochloride (TC) concentrations were analyzed by Jena uv-vis-vis spectrometer (SPECCORD200PLUS) at 356 nm.Spectrophotometry was used to analyze the concentration of Fe and Co in the color developing solution at 510 nm by the absorbance of the color developing solution using 1, 10-phenanthroline^29,30^ and 5-cl-padab^31,^ respectively. The total organic carbon was determined by a Multi 3100 TOC/TN analyzer (Analytik Jena AG).For the TOC analysis test, all samples were immediately treated with scavenging agents (0.05 M Na_2_SO_3_, 0.05 M KH_2_PO_4_, 0.05 M KI and 0.02 M NaOH) to obtain accurate TOC values^14,32^.

The sample structure was qualitatively analyzed by field emission scanning electron microscope FE-SEM; Hitachi S-4700 II at 15.0 kV accelerated voltage, JEOL JEM 2010 F electron microscope at 200 kV and physical D/max-2400X-ray diffractometer with Ni filter Cu K^+^ radiation, respectively. Micromeritics ASAP 2020 was used to analyze the sample surface area and pore diameter distribution by N_2_ adsorption and desorption, and the sample surface area was calculated by Brunauer_Emmett_Teller (BET) method.

## Results and Discussion

### Morphology and structure

**Figure 1:**
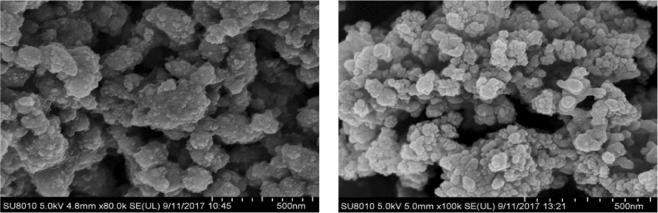
SEM images of meso-Fe/Co.

**Figure 2:**
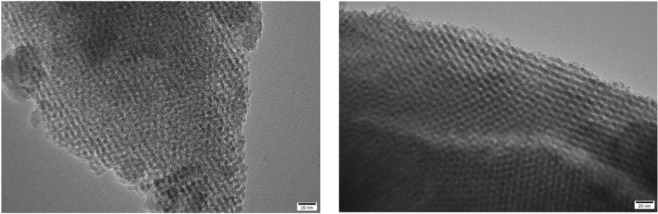
TEM images of meso-Fe/Co.

**Figure 3:**
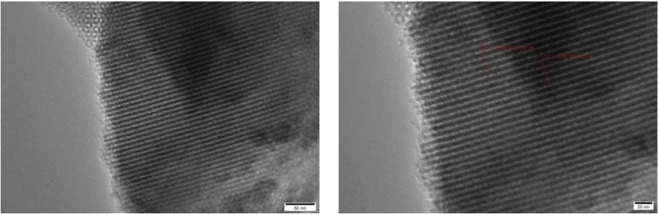
TEM images of KIT-6.

**Figure 4:**
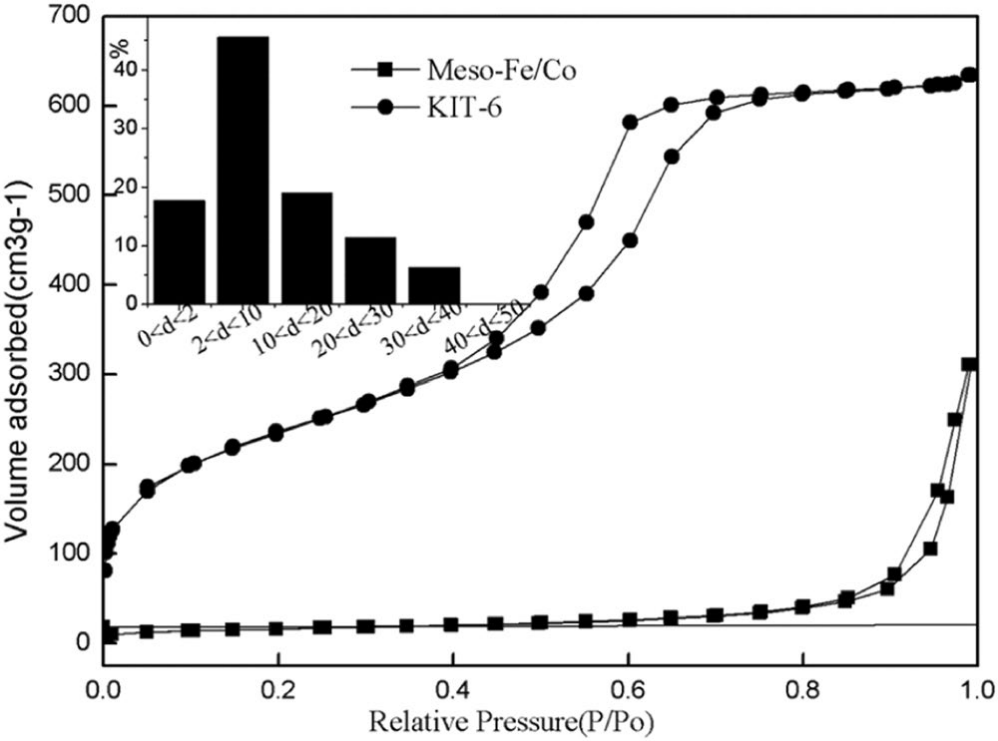
N_2_ adsorption–desorption isotherms and pore size distribution (inset) of meso-Fe/Co.

The characterization of catalysts helps to a better understanding of the relationship between catalytic properties of catalysts and their physical structures. SEM and TEM images clearly show perfect micropores and physical structure, uniform shape and smooth surface. In order to further study the mesoporous structure of the catalyst, the sample was described. According to the analysis of the sample SEM image (Fig. 1) and TEM image (Figs 2, 3), the catalyst effectively entered the intermediate pore structure of the template during the preparation process. According to the statistical analysis, the catalyst sample is composed of spherical particles of different sizes, and the catalysts between particles are mainly distributed in the range of 2 to 10 nm.The aperture of the KIT-6 was about 6.8 nm (Fig. 4), which was consistent with the results of previous studies^12^.

**Figure 5:**
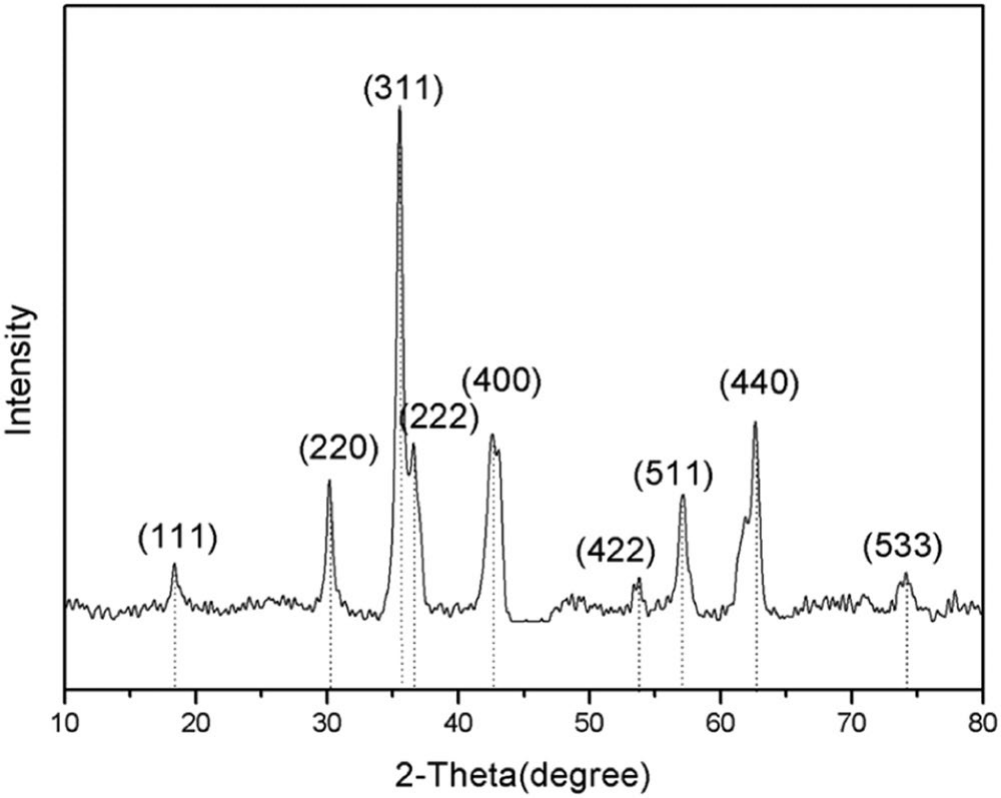
Wide-angle XRD patterns of meso-Fe/Co.

XRD analysis of Mes -Fe/Co shows that phase analysis of catalyst according to PDF card 22–0864#, 22-1086#. In 2T = 35.1° and 41.4° and 50.4° and 62.9° and 67.3° and 74.2° and 88.6° there is obvious diffraction peak, corresponding alloy (220) (311) (222) (400) (422 (511) (440) and (533) plane^33^(Fig. 5), showed that the method is used to Fe/Co bimetallic catalyst was successfully achieved. The position and relative strength of the diffraction peak of the synthetic bimetallic Fe/Co catalyst are consistent with the standard XRD data of the spinel (No. 22-1086 and No. 03-0864).

The results of N_2_ adsorption-desorption isotherm study on the specific surface area and porosity of samples show that the adsorption isothermal curve of the sample is H3 hysteresis loop IV (Fig. 4), which is a typical mesoporous characteristic structure with a specific surface area of 135.8 m^2^ g^−1^, much higher than that of commercial Fe_3_O_4_ (~2 m^2^g^−1^)^34^.The results of pore diameter of catalyst by Barrett- Joyner-Halenda (BJH) method showed that the pore diameter of the catalyst is mainly in the range of 1–40 nm, about 40% pore is distributed in the range of 2–10 nm mesoporous^35^, and about 20% pore size is distributed in the range of 10–20 nm.

Studies have shown that heterogeneous catalysts with rich pore diameter and large specific surface area have excellent catalytic activity^33–35^. Abundant pore diameter structure is not only conducive to increasing the specific surface area of the catalyst and the catalytic activity point of the catalyst, but also reducing the transfer resistance of pollutants in the pore diameter. At the same time, the catalyst has a good adsorption performance to promote the heterogeneous Fenton reaction process. The specific surface area of Mes-Fe/Co was 135.8 m^2^ g^−1^, and the pore diameter was mainly distributed in the range of 1–40 nm. About 40% of the pore diameter was distributed in the range of 2–10 nm mesoporous, and about 20% was in the range of 10–20 nm. It is stated that the catalyst has not only a rich pore diameter structure, but also a large specific surface area indicates that the catalyst should have excellent catalytic performance.

### The performance of catalysts

#### Catalytic activity analysis

UV spectrophotometric analysis of the sample helps to understand the intensity and length of time produced by •OH, a large amount of TC was degraded and transformed, which indirectly indicated that the catalyst could catalyze the production of •OH for a long time. However, its production is not enough to completely degrade and mineralize the degraded and transformed TC. A large amount of TC was mineralized, indicating that the catalyst produced enough •OH in a certain period of time. Not only can TC be degraded, but also the degradation products can be further degraded and finally mineralized effectively

**Figure 6:**
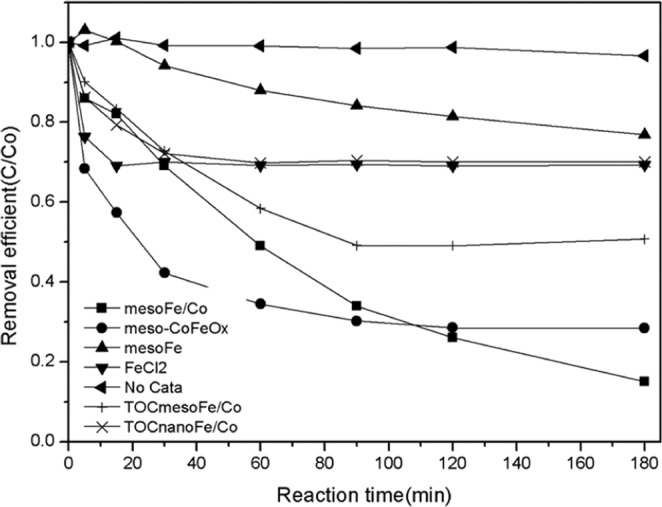
Performance comparison of different catalysts.

The experiment was carried out with catalysts under the following conditions: Mes-Fe/Co = 0.6 g/L, H_2_O_2_ = 30 mmol, TC = 30 mg/L, pH = 7, T = 25.5 °C (Fig. 6). Under the same experimental conditions, mes-Fe/Co has the best catalytic activity, which is better than any single element. Its catalytic activity is 2.7 times that of iron ion and 3.6 times that of mesoporous iron. The mineralization rate of TC in 90 minutes was over 50%, the TC degradation rate in 1.8 hours was 70%, and the TC degradation rate in 3 hours was 86%, which was similar to the result 36 reported previously^36,37^.

#### Effect of iron-to-cobalt ratio

**Figure 7:**
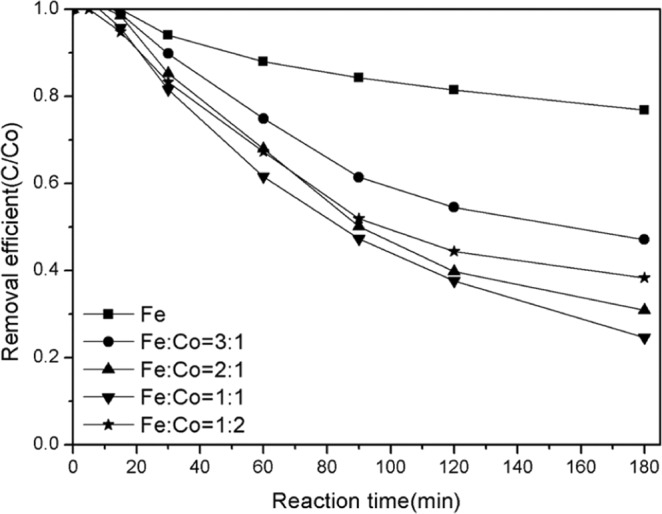
Effect of Fe/Co ratios on catalyst performance.

The results show that the synergistic effect of two or more metals can improve the activity of the catalyst. In order to better understand the role of cobalt in bimetallic catalysts, a series of comparative experiments were conducted (Fig. 7). The results showed that the Fenton activity at the catalyst site not only increased significantly with the increase of cobalt content, but also the reaction speed of Fenton. However, when the content of cobalt increased to a certain value, the activity of the catalyst decreased. The best Fenton properties were found in catalysts with Fe/Co molar ratios of 2:1 and 1:1.This shows that the co-action between iron and cobalt is more favorable near the 1:1 ratio of iron and cobalt.

#### Effect of pH

**Figure 8:**
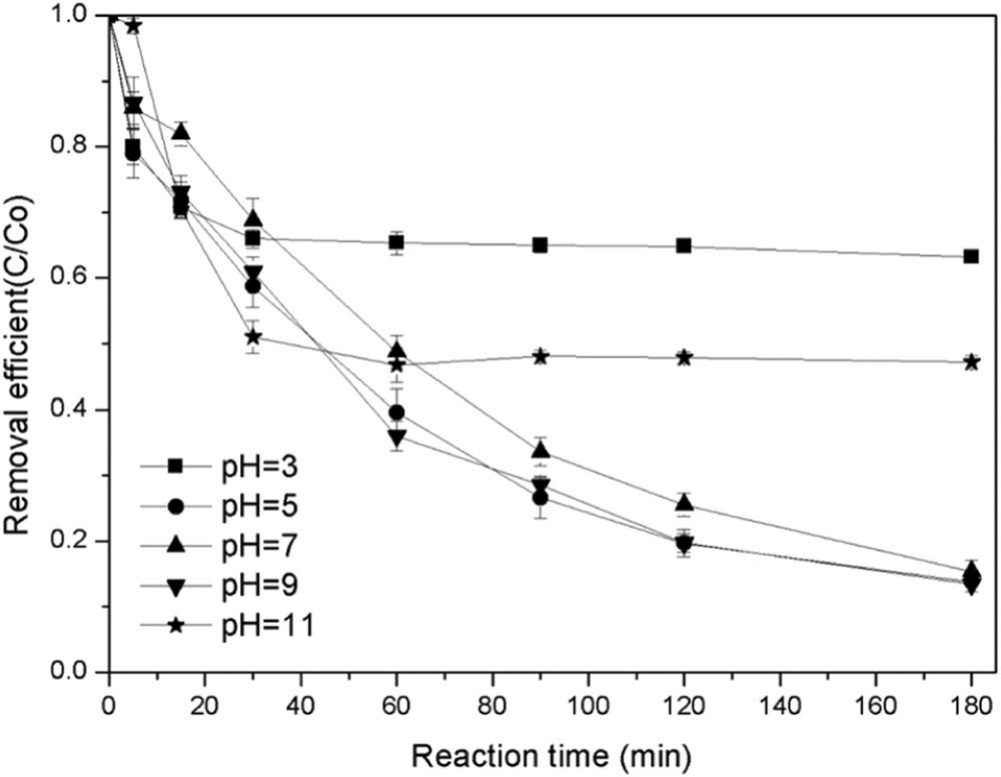
Effect of pH on catalyst performance.

**Figure 9:**
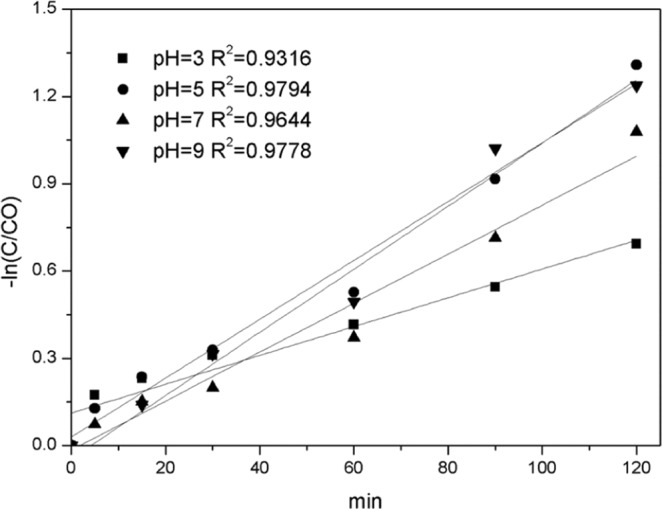
Pseudo first-order dynamic analysis of TC removal under different pH conditions.

Since most discharges have an acidic or alkaline pH, the optimal pH for most chemical, physical, and biological reactions is near neutral. The pH values before and after the reaction are often adjusted to ensure pollutant removal and wastewater discharge requirements. Therefore, the study on highly active Fenton-like catalyst in different pH (Fig. 8) has an obvious usage prospective and economic value. The results reveal that the removal efficiency of catalyst at pH= 5~9 was better than that of catalyst at pH = 3 and 11. The removal rate of TC at pH 5~9 can reach 90%. indicating that bimetallic Fe/Co can degrade TC in a larger pH range. Pseudo-first-order reaction kinetics can better explain this process (Fig. 9). At a pH of 5, 7 and 9, the removal rate constants (k) were 0.01, 0.084 and 0.011 min^−1^, respectively, which were 2.1, 17.8 and 2.3 times of these at a pH of 3 (0.0047), respectively. However, removal efficiency is also relatively low at a high pH. Literature reported that H_2_O_2_ does not produce hydroxyl group at high a pH^21,38^. The pseudo-first-order model can well explain the degradation kinetics curve of TC, as shown below^11^:

$$-\,\mathrm{ln}({\rm{C}}/{{\rm{C}}}_{0})={\rm{kt}}+{\rm{b}}$$

where k is the pseudo first-order rate constant, C_0_ is the initial concentration, and C is the concentration at time t.

#### Effect of H_2_O_2_ concentration

**Figure 10:**
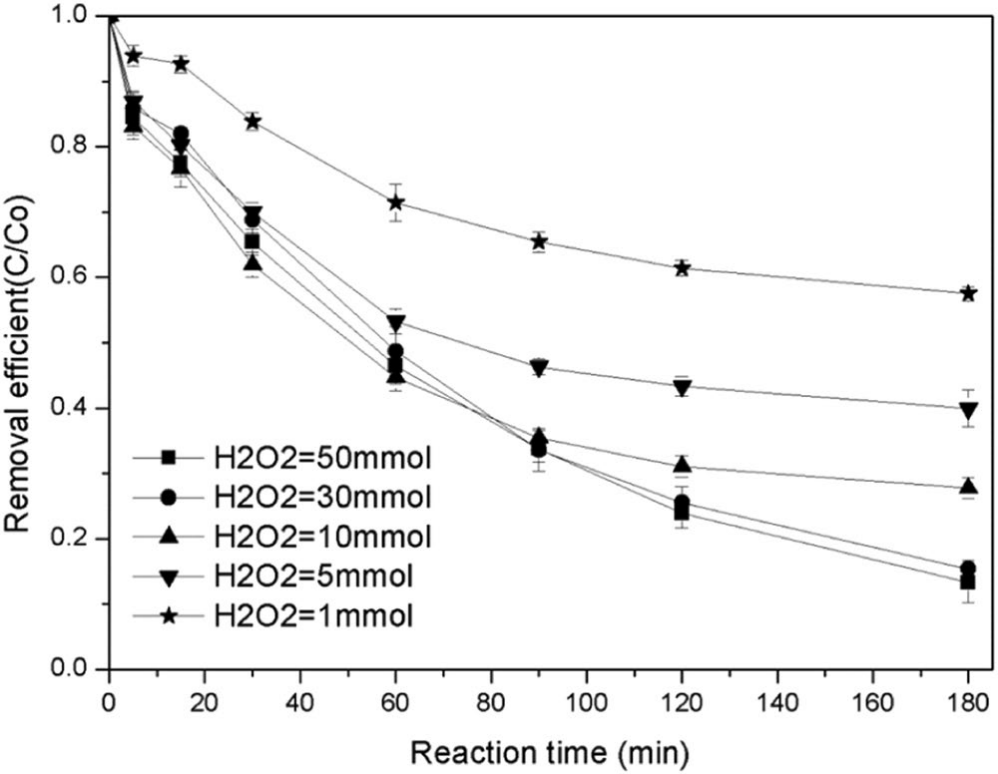
Effect of H_2_O concentration on catalyst performance.

**Figure 11:**
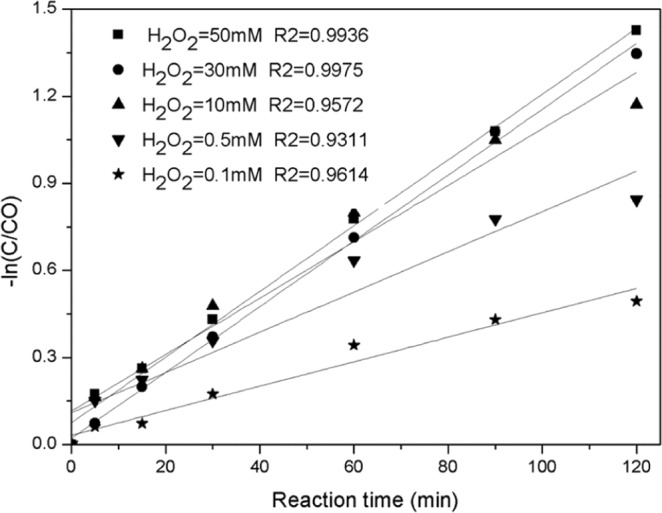
Pseudo first-order dynamic analysis of TC removal under different H2O2 conditions.

Hydroxyl radicals are produced from hydrogen peroxide, which acts as the actual oxidant for the removal of TC. The amount of H_2_O_2_ has a direct effect on the mineralization of TC degradation. The results showed (Fig. 10) that TC degradation efficiency increased from 42.5% to 86.7% with increasing H_2_O_2_ concentration from 1 to 50 mmol. However, when the content of H_2_O_2_ exceeds 30 mmol, the degradation efficiency of TC has no obviously increases. According to the pseudo-first-order reaction kinetics, the k value of TC degradation efficiency at 30 mmol was 1.6 times higher than that at 10 mmol (Fig. 11). The results suggested that high H_2_O_2_ concentration can promote effective contact with the catalyst and promote the reaction of •OH and TC within a certain concentration range (Eq. 1, 2). However, if the concentration of H_2_O_2_ is too high (>30 mmol), the reaction of •OH with H_2_O_2_ and •O_2_H may be promoted (Eq. 3, 4), leading to the scavenging effect of •OH^39–41^.

1 $${{\rm{Fe}}}^{2+}+{{\rm{H}}}_{2}{{\rm{O}}}_{2}\to \bullet {\rm{OH}}+{{\rm{Fe}}}^{3+}{+}^{-}{\rm{OH}}$$

2 $${{\rm{Fe}}}^{3+}+{{\rm{H}}}_{2}{{\rm{O}}}_{2}\to {{\rm{Fe}}}^{2+}+\bullet {{\rm{O}}}_{2}{\rm{H}}+{{\rm{H}}}^{+}$$

3 $$\bullet {\rm{OH}}+\cdot {\rm{OH}}\to {{\rm{H}}}_{2}{{\rm{O}}}_{2}$$

4 $${{\rm{H}}}_{2}{{\rm{O}}}_{2}+2\cdot {\rm{OH}}\to 2{{\rm{H}}}_{2}{\rm{O}}+{{\rm{O}}}_{2}$$

#### Influence of pollutant concentration on catalyst performance

**Figure 12:**
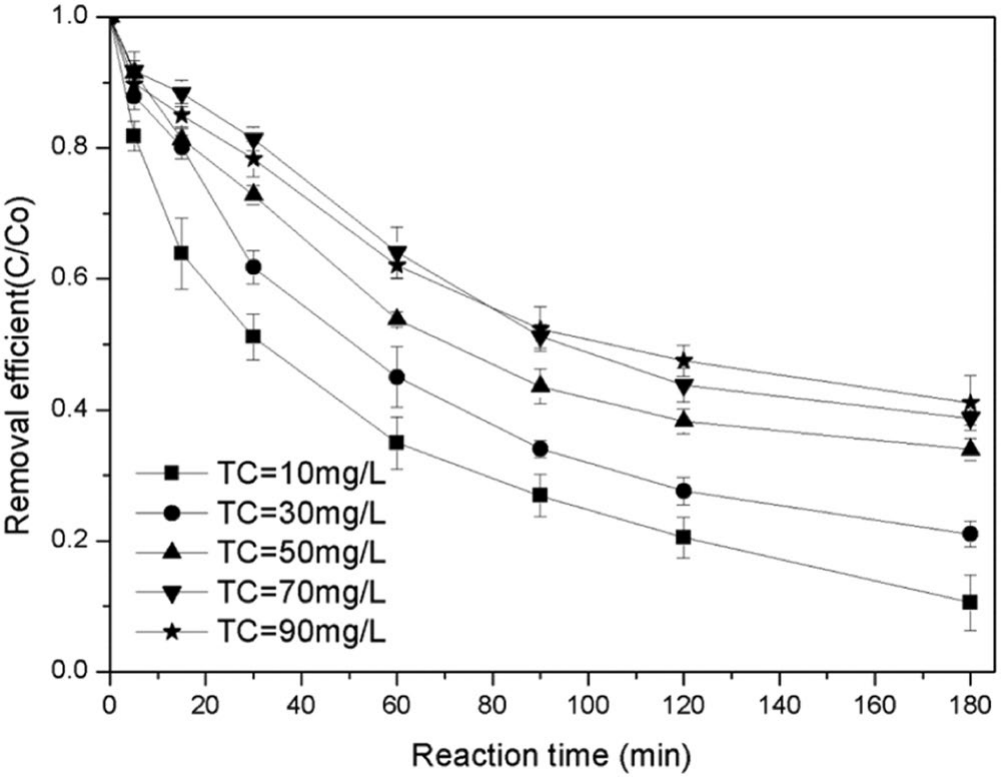
Effect of TC concentration on catalyst performance.

**Figure 13:**
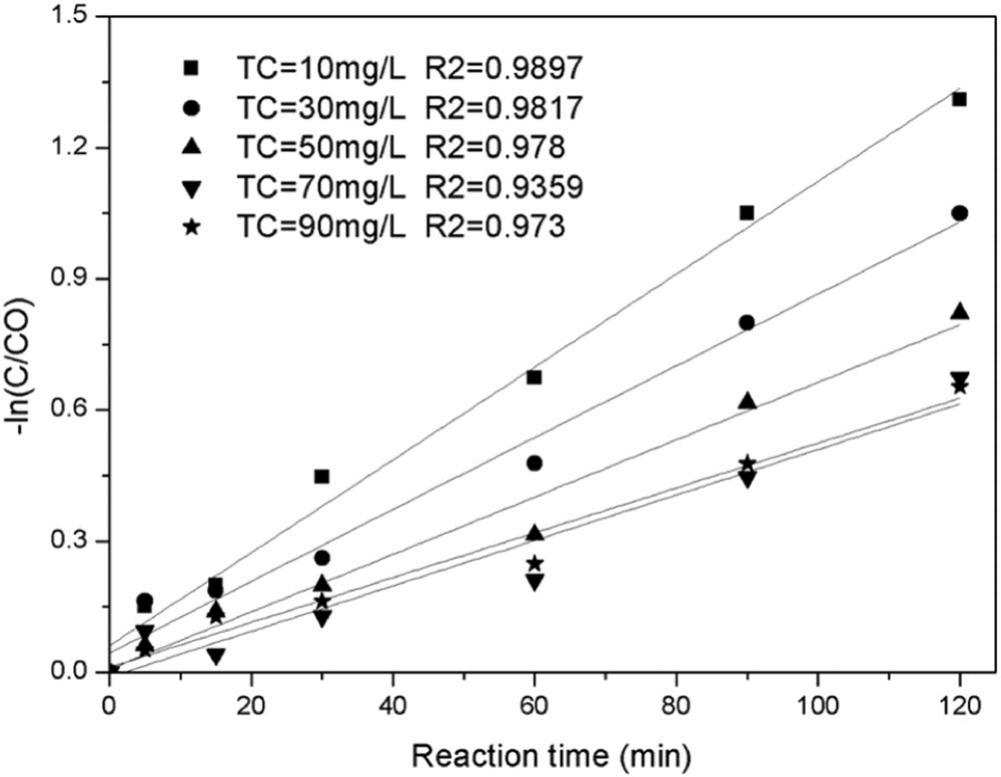
Pseudo first-order dynamic analysis of TC removal under different TC conditions.

The removal rate of TC gradually decreases with an increase in pollutant concentrations (Fig. 12). The removal rates of TC ranged from 89.4% to 58.92% when the concentrations of TC ranged from 10 mg/L to 90 mg/L. In the first five minutes after the reaction began, higher reaction rates were observed at all concentration levels because of the maximum values of H_2_O_2_ and the contaminant. The generation rate of free radical and its probability of exposure to the contaminants were at the maximum level. However, with the consumption of pollutants and H_2_O_2_, the production of free radicals decreased, as well as the probability of exposure to TC; thus, the degradation reaction of TC was mild. According to the first-order reaction model (Fig. 13), the reaction rate decreases with an increase in the concentration of the pollutant, but the degradation rate does not change with pollutant concentration when the pollutant concentration is higher than 50 mg·L^−1^. This is because the catalyst surface area and the amount of H_2_O_2_ are the main factors limiting the production of free radicals at high concentrations. Therefore, low concentrations are conducive to the degradation and mineralization of pollutants, whereas high concentrations are more conducive to the efficient use of H_2_O_2_ and the decomposition of pollutants into small molecules.

### Catalytic stability of meso-Fe/Co

**Figure 14:**
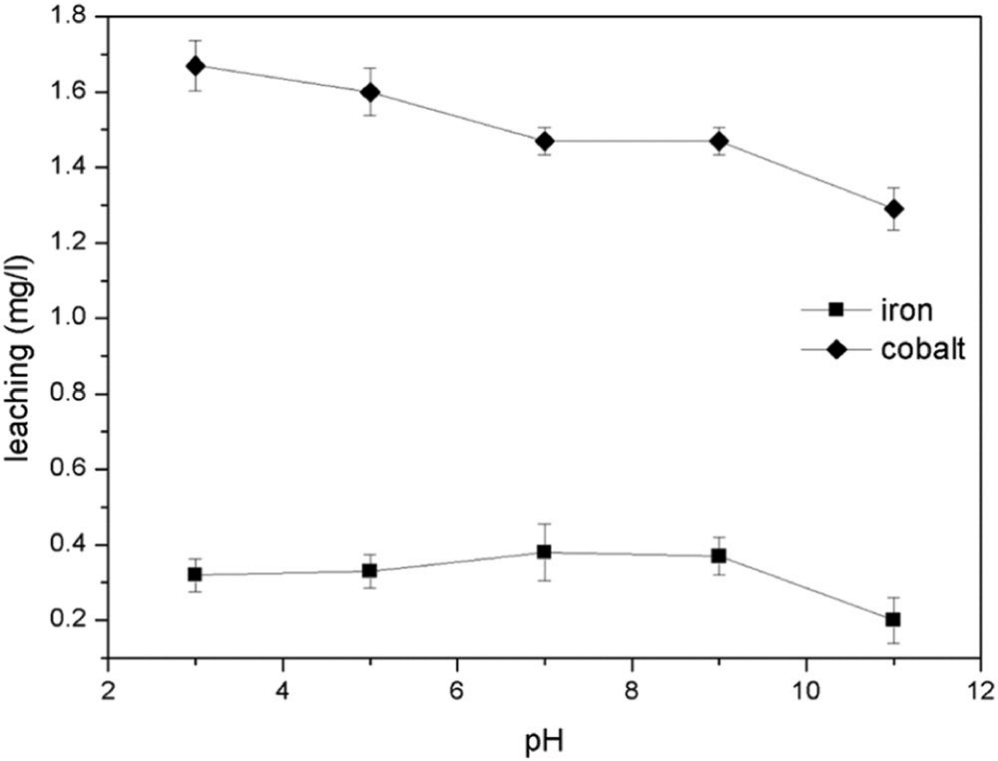
Concentration of iron leaching in solution during TC degradation in 3 h.

Successive experiments were conducted to evaluate the catalyst stability. Previous studies showed that when solution is acidic, the concentration of H^+^ in the Fenton-like reaction are very high; This is not conducive to the stability of the catalyst and can accelerate the dissolution of active components iron and cobalt^38^. After 180 min of reaction under different pH values (from 3 to 11), the precipitation of cobalt gradually decreased (1.66 mg/L, 1.6 mg/L, 1.5 mg/L, 1.5 mg/L, 1.3 mg/L) whereas the precipitation of iron was not affected by pH (0.31 mg/L, 0.33 mg/L, 0.36 mg/L, 0.37 mg/L), although it was 0.20 mg/L at pH 11(Fig. 14). These samples had lower leaching degrees than nano-Fe3O4 (2.3 mg/L) and Fe_3_O_4_ (9.8 mg/L after 180 min) as reported previously^30^. The leaching of Fe ions is kept below 1 mg/L, which is acceptable according to the European Union and the United States discharge standards (<2 ppm)^42^. The cobalt ion leaching also meets China’s Environmental Quality Standards for Surface Water (<2 ppm) (GB 3838-2002). Thus, the treated pollutants can be discharged directly without worrying about heavy metal pollution, which is beneficial from the perspective of long-term ecological environment protection and development.

**Figure 15:**
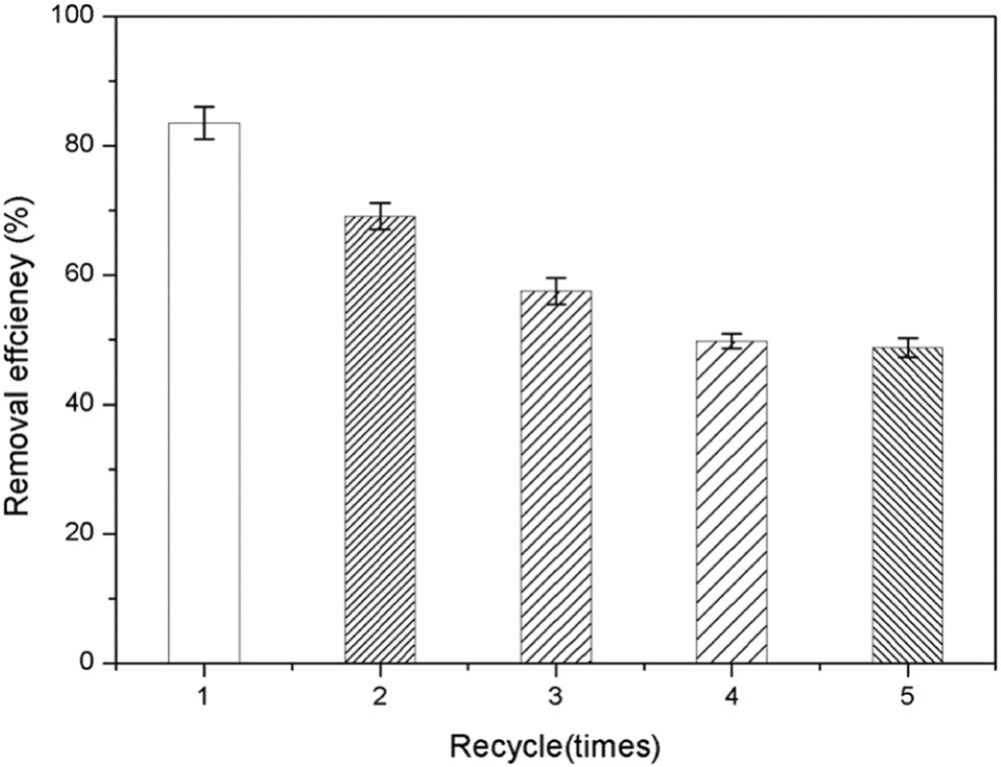
Catalytic activity of meso-Fe/Co composite in different cycling numbers.

Catalytic reactions are repeated many times in succession, the activity of the catalyst decreased gradually after four successive catalytic degradation reactions (Fig. 15). Possible reasons for the decrease of catalyst activity:1) Fe and Co ions on the surface of the catalyst form stable complexes with ^−^OH in the solution, leading to the decrease of the number of active sites on the surface of the catalyst; 2) the active components Fe and Co at the active site of the catalyst were dissolved from the surface. This study revealed that catalyst deactivation has many factors, including reduced catalyst surface area, catalyst poisoning, and active ingredient dissolution. Research is needed to further explore these factors.

### Potential mechanisms

**Figure 16:**
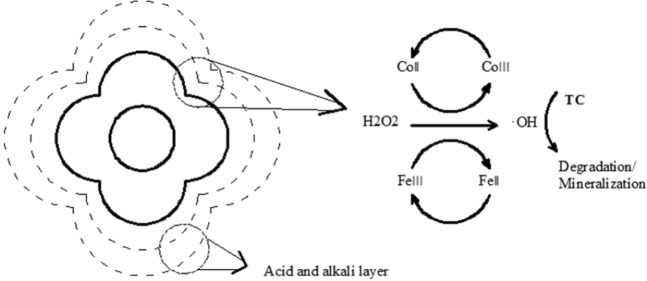
Schematic illustration of the reaction mechanism of H_2_O_2_ activation by meso-Fe/Co.

In general, the pH required for the Fenton or Fenton-like reaction is about 3, and when the pH of the reaction solution is higher than 6, the Fenton or Fenton-like reaction is not excited. In this study, the reaction solution is still active when exceeding a pH of 9. Possible reasons for this phenomenon follow as: (1) Co has a Fenton property similar to Fe^2+^, which can activate H_2_O_2_ to generate free radicals under a wide range of acid-base conditions (Eqs 5, 6)^39^ (Fig. 16), so that the Fenton reaction can continue^5^^,^^43–45^; (2) Co has Lewis acidity, which can form an acid-base complex as an electron pair acceptor and a hydroxide electron pair in the solution, forming an acidic or slightly acidic environment around the catalyst^46,47^. The hydroxide stability of Co is stronger than that of iron Fe^48,49^. Meanwhile, the hydroxide stability can reduce the deactivation amount of Fe element and promote the Fenton or Fenton-like reaction of Fe element.

5 $${{\rm{Fe}}}^{2+}+{{\rm{H}}}_{2}{{\rm{O}}}_{2}\to {{\rm{Fe}}}^{3+}+\cdot {\rm{OH}}+2{{\rm{H}}}_{2}{\rm{O}}\,({\rm{acidic}}\,{\rm{pH}})$$

6 $${{\rm{Co}}}^{2+}+{{\rm{H}}}_{2}{{\rm{O}}}_{2}\to {{\rm{Co}}}^{3+}+\cdot {\rm{OH}}+{{\rm{OH}}}^{-}$$

7 $${{\rm{Co}}}^{2+}+{{\rm{H}}}_{2}{\rm{O}}\to {{\rm{CoOH}}}^{+}+{{\rm{H}}}^{+}$$

8 $${{\rm{Fe}}}^{3+}+{{\rm{H}}}_{2}{\rm{O}}\to {{\rm{FeOH}}}^{2+}+{{\rm{H}}}^{+}$$

9 $${{\rm{Co}}}^{2+}+{{\rm{FeOH}}}^{2+}\to {{\rm{CoOH}}}^{+}+{{\rm{H}}}^{+}$$

## Conclusions

The Meso-Fe/Co heterogeneous Fenton catalyst was successfully prepared by template method. Qualitative and quantitative characterization of meso-Fe/Co catalyst by TEM, SEM and BET showed that meso-Fe/Co catalyst has not only a large specific surface area 113.8 m^2^g^−1^, but also a large number of mesoporous pore diameter distribution. Catalytic performance and operating parameters show that meso-Fe/Co catalysts have a satisfactory removal effect on TC (86%), a good stability (precipitation cobalt and iron were less than 1.66 mg/L and 0.33 mg/L) and a wider range of pH use (good removal efficiency in 5–9 range). Therefore, the high catalytic efficiency, a smaller amount of leached ions, a wider pH application range of bimetallic Fe/Co and its convenient recycling without any regeneration made the catalyst attractive for potential applications. Unfortunately, the XPS characterization and the generation of catalyst microenvironment were not verified by corresponding experiments due to objective factors and experimental conditions.
